# Clinical-Grade Human Pluripotent Stem Cells for Cell Therapy: Characterization Strategy

**DOI:** 10.3390/ijms21072435

**Published:** 2020-03-31

**Authors:** Daniela Rehakova, Tereza Souralova, Irena Koutna

**Affiliations:** 1 Department of Experimental Biology, Faculty of Science, Masaryk University, Kamenice 5, 625 00 Brno, Czech Republic; danka.rehakova@mail.muni.cz; 2 International Clinical Research Center, St. Anne’s University Hospital Brno, Pekařská 53, 656 91 Brno, Czech Republic; souralova@med.muni.cz; 3 Department of Histology and Embryology, Faculty of Medicine, Masaryk University, Kamenice 3, 625 00 Brno, Czech Republic

**Keywords:** hPSCs, human pluripotent stem cells, characterization, hESC, human embryonic stem cells, hiPSC, human induced pluripotent stem cells, clinical, cGMP, cell therapy

## Abstract

Human pluripotent stem cells have the potential to change the way in which human diseases are cured. Clinical-grade human embryonic stem cells and human induced pluripotent stem cells have to be created according to current good manufacturing practices and regulations. Quality and safety must be of the highest importance when humans’ lives are at stake. With the rising number of clinical trials, there is a need for a consensus on hPSCs characterization. Here, we summarize mandatory and ′for information only′ characterization methods with release criteria for the establishment of clinical-grade hPSC lines.

## 1. Introduction

Human pluripotent stem cells (hPSCs) have held great promise for regenerative medicine since derivation of human embryonic stem cells by Thompson in 1998 [1] and the first establishment of human induced pluripotent stem cells (hiPSCs) by Takahashi and Yamanaka in 2007 [2]. Both hESCs and hiPSCs are able to self-renew, express the same human pluripotent markers and differentiate into all three germ layers—ectoderm, mesoderm and endoderm [2]. Their proliferative and differentiation capacities are highly convenient for cell substitution therapy because they enable the propagation of cells to obtain their required amounts and the possibility of creating any cell type from the human body. In these aspects, they are superior to other stem cells—such as mesenchymal stem cells or hematopoietic stem cells—which have only limited proliferation potential and can only differentiate into a few specific cell types [3,4].

The main difference between hESCs and hiPSCs is in the way they are created. While hESCs are derived from the inner cell mass of human preimplantation blastocysts which are not suitable for in vitro fertilization (IVF) [1], hiPSCs are reprogrammed from human somatic cells. The first raises ethical concerns as human blastocyst is used in order to obtain hESCs. The second one raises uncertainty as reprogramming associated DNA methylation, which enables us to distinguish hiPSCs from hESCs, is not yet fully understood [5,6,7]. Furthermore, persistent donor cell gene expression after somatic cell reprogramming was also observed [8,9]. It was suggested that incomplete DNA methylation is the basis of transcriptional memory of somatic cells in hiPSCs [10]. Nevertheless, hiPSCs are freed from the ethical burden and can be prepared from various types of somatic cells [2,11,12]—in comparison with hESCs, that have only one biological source.

Treatment with hiPSCs in an autologous setting is still associated with a high medical cost [13] as it was estimated that production of clinical-grade autologous hiPSCs in compliance with cGMP would cost approximately USD 800,000 in comparison with USD 10,000 to USD 20,000 that would cost to prepare research-grade hiPSC line alone [14]. Luckily, hiPSCs can be used in an allogeneic setting, similarly to hESCs. As with for tissue and organ transplantation, the donor-recipient match for allogeneic treatment has to be as accurate as possible. Based on these facts, there is an effort to establish hiPSC and hESC banks for HLA-matched tissue transplantation [15,16,17], in order to provide hPSC-based therapy to a maximal number of patients.

While some discuss the use of undifferentiated hiPSCs as a therapy product [18,19], the majority of hPSCs applications assume their directed differentiation before use, since undifferentiated cells can pose a risk of tumorigenicity. Many different cell types have been generated from hPSCs and some have already been used in clinical trials.

Since hESCs were the first type of hPSCs established, it is not surprising that the first hPSCs-in-humans clinical trial was conducted using hESCs-derived cells. More specifically, Geron conducted a safety study of hESCs-derived oligodendrocyte progenitor cells for spinal injury treatment in 2010 [20]. While the study was terminated early, five patients were treated with no adverse effect. Based on this trial, a dose-escalated study was started (NCT02302157) by Asterias Biotherapeutics, Inc. in 2015. The results seem promising, as no adverse effects were observed, and motor function improved in some cohorts [21]. Clinical trials have been opened to treat heart failure with hiPSCs-derived cardiomyocytes [22,23].

Some clinical studies focused on the utilization of hPSCs in the treatment of eye disorders, more specifically macular degenerations. The first in-human hiPSCs clinical study (even if it was stopped) used an implanted autologous hiPSCs-derived sheet of retinal pigment epithelial cells in a patient with neovascular age-related macular dystrophy [24]. In 2012, two clinical studies using hESC-derived retinal pigment epithelium were started by the Astellas Institute for Regenerative Medicine [25]. Recently a clinical trial using hiPSCs-derived corneal epithelial cell sheet transplantation for patients with limbal stem-cell deficiency was started [26]. Focusing on eye treatment in the early stages of clinical testing has several advantages including the blood–ocular barrier separating the eye from the rest of the organism (enhancing safety and reducing the risk of immune rejection), and the easy non-invasive evaluation of the outcome.

As described above, multiple clinical trials using hPSC-derived cells have been launched, and data from some of them are already available. The therapeutic potential of hPSCs is tremendous and great progress has been made from bench to bedside since their first derivation, but there are still some hurdles that need to be overcome. One of them is a need for standardization. The most feasible way to benefit from hPSCs is to make a bank or multiple banks of clinical-grade hPSC lines available for further processing in specific application. Since we need to be able to compare the banked cell lines, coming to a consensus on their quality assessment is of utmost importance. On that account, we present an overview of characterization strategies used so far in the derivation of clinical-grade hPSCs and propose a set of mandatory tests including release criteria and ′for information only′ tests.

## 2. Requirements for the Manufacture of Clinical-Grade hPSCs

### 2.1. Current Good Manufacturing Practices

In order to translate hPSCs into clinical practice, it is necessary to manufacture and perform quality control of hPSCs according to current good manufacturing practices (cGMP). cGMP are defined by the European Medicines Agency (EMA, https://www.ema.europa.eu/en) in the European Union and by the US Food and Drug Administration (FDA, https://www.fda.gov/) in the USA. Briefly, cGMP require that medicines a) are of consistent high quality; b) are appropriate for their intended use; c) meet the requirements of the marketing authorization or clinical trial authorization [27].

According to cGMP, the establishment of clinical-grade hPSCs requires the implementation of a quality control system and the consistent documentation of the whole process—from the procurement of biological material, culture and freezing to quality control (QC) testing and distribution. Every step of this process has to be validated and executed according to standard operating procedures (SOPs). SOPs ensure that the reproducibility of the final product is in accordance with established specifications [28]. The use of hPSCs in the clinic has to be evaluated according to the release criteria of a clinical trial to ensure their suitability for the intended purpose.

hPSC culture also has some specifics that need to be implemented into quality control. It is well-known that hPSCs acquire various genetic changes connected with culture conditions and time in culture [29,30]. As these genetic aberrations could be harmful, it is necessary to verify the genetic stability of manufactured clinical-grade hPSCs lines. Furthermore, it is absolutely essential to repeatedly perform sterility tests during culture and, of course, to test the final product for any contamination.

As hPSCs derivates should be used in replacement therapies, i.e., in direct contact with a human body, it is essential to ensure that animal-derived molecules do not cause an immune reaction to a patient. It was found that hESCs express culture-acquired immunogenic nonhuman sialic acid (Neu5Gc), a component of animal-derived serum replacement and animal feeder cells [31]. As many humans have antibodies against Neu5Gc, human immunoglobulin binds to hESC-derived cells, which eventually leads to specific cell killing [31]. The presence of viruses in mouse embryonic fibroblasts, commonly used as feeders in the research area, was also observed [32]. Therefore, xeno-free and defined media and surfaces for culture are developed and used for the establishment of clinical-grade hPSC lines [33,34].

### 2.2. Regulations

The establishment of hPSC lines must be done in accordance with the relevant laws and policies of the country where the derivation is performed. Information on the current legal position, ethical and regulatory oversight for EU countries can be found on https://www.eurostemcell.org. However, there are directives and guidelines in force for all EU countries that specify good manufacturing practices relevant for the establishment of hPSCs that must be obeyed. The main directives and guidelines relevant to the establishment of clinical-grade hPSC lines are summarized in Table 1.

Similarly, hPSC lines derived in the USA must be compliant with federal regulations and FDA regulations. Because stem cell research and translation to clinical practice are moving very fast, it is recommended to search for the current regulations and directions relevant to your country.

Worldwide, there are two international organizations focusing on the field of human pluripotent stem cells: The International Stem Cell Forum (ISCF, http://www.stem-cell-forum.net/) and the International Society for Stem Cell Research (ISSCR, https://www.isscr.org/). The ISSCR released guidelines for stem cell research and clinical translation in 2016, that can provide useful information about the ethics, research and clinical translation of hPSCs.

## 3. Characterization Methods

### 3.1. Markers of Human Pluripotent Stem Cells

hPSCs can be distinguished by a set of markers that relate to their pluripotent status. Among the most known pluripotency markers belong the transcription factors OCT3/4, SOX2 and NANOG, tumor rejection antigens TRA-1-60, TRA-1-81, and stage-specific-embryonic antigens SSEA3 and SSEA4 [35]. It is worth mentioning that hPSCs are negative for the SSEA1, marker of pluripotency for murine pluripotent stem cells [36]. In humans, SSEA1 is expressed during differentiation, therefore hPSCs should be negative for SSEA1.

#### 3.1.1. Flow Cytometry

Flow cytometry has been extensively used for the detection of various cellular markers. Surface markers (TRA-1-60, TRA-1-81, SSEA3, SSEA4) are easier to detect as antigens are accessible for antibodies. The protocol for the detection of intracellular markers (OCT3/4, SOX2, NANOG) has an additional step enriched with fixation. A combination of surface markers and intracellular markers is favored. This method is fast and robust, and the results are quantitative; therefore, it is easy to compare them in time and amongst different laboratories.

The characterization of intracellular markers has been used by many authors for the establishment of clinical-grade hESC lines [33,37,38] and hiPSC lines [11]. There is no consensus on release criteria as these differ in published protocols for the establishment of clinical-grade hPSCs. However, there are efforts to determine the required number of markers and their thresholds to meet specifications for clinical-grade hPSC lines [39,40,41]. The release criteria from published papers of successful derivations of hPSCs are summarized in Table 2.

#### 3.1.2. Immunocytochemistry

Immunocytochemistry is a laboratory assay for the detection and localization of various cell components. It is based on primary and secondary antibody binding. The secondary antibody is connected to a fluorophore that allows detecting the signal under a fluorescence microscope. The advantage of this method is that in addition to the presence of the antigen, we can also evaluate its localization within the cell. On the other hand, interpretation is difficult; thus, comparability within other results can be problematic. Other points to consider are that this technique requires trained personnel, negative controls and strict evaluation criteria.

Both surface (TRA-1-60, TRA-1-81, SSEA3, SSEA4) and intracellular markers of pluripotency (OCT3/4, SOX2, NANOG) can be detected by immunocytochemistry. A combination of surface markers and intracellular markers is favored, although immunocytochemistry is recommended to be used only for informative purposes according to The International Stem Cell Initiative for hESCs [41] and guidelines for the characterization of hiPSCs [40].

Immunocytochemistry was used for the detection of pluripotent markers of clinical-grade hESC lines by some authors. The determination of thresholds is difficult; nevertheless, the cited authors published their release criteria as you can see in Table 3 [43,44].

#### 3.1.3. Alkaline Phosphatase

As an early marker of the successful establishment of a hPSC line, alkaline phosphatase (AP) staining is often performed [45]. hPSCs express high amounts of AP, which can be visualized by a chromogenic substrate [46]. The test is quick and easy to perform, the positivity of colonies is examined under a microscope, but is visible to the bare eye as well.

### 3.2. Genetic Testing

#### 3.2.1. Fingerprinting

The distinction of various hPSC lines is crucial not only for experimental purposes but mainly in clinical usage. There is a risk of unwanted switch or cross-contamination between cultured hPSC lines that can be life-threatening in the case of directed differentiation and subsequent transplantation to humans [47]. Identification of the particular hPSC line has to be unambiguous, therefore short tandem repeats (STR) loci are analyzed. There are numerous commercially available kits for cell line authentication [41,48], although for a few STR profiles establishment is worth consideration to send DNA samples to an accredited laboratory [37].

The STR profile should match the cell donor in the case of the establishment of a hiPSC line, but this connection is impossible to establish for hESC lines as there are not many cells at the time of derivation. However, it is recommended to establish the STR profile in early passages, as it can help to recognize unwanted switch or cross-contamination.

At least 8 core STR loci with an 80% threshold match should be used according to ANSI/ATCC ASN-0002-2011 standard for the authentication of human cell lines. However, 15 STR loci may be required for the unambiguous identification for autologous usage of hPSCs [49].

Eight specific loci plus amelogenin were used for the STR profiling of clinical-grade hESCs by De Sousa [42], and 16 specific loci were used for the establishment of clinical-grade hESC lines by Tannenbaum [33]. Sixteen STR specific sites were used for the STR profile of hiPSCs established according to cGMP [11].

#### 3.2.2. Karyotype

Karyotype analysis is a standard technique for the determination of genome stability. hPSCs are often unstable during long-time culture [50,51]; therefore, it is recommended to verify genetic integrity of hPSCs before further processing (creating master cell bank, differentiation to transplantable cells, etc.).

Giemsa-banding (G-banding) should be used for chromosome counts of 20 metaphases, and further banding patterns analysis in a minimum of 8 metaphases [41]. This testing confirms with a 95% probability that tested cells are karyotypically normal. If there are some hPSCs with abnormalities, repeat testing is recommended, as there could be a population of karyotypically abnormal hPSCs [39]. It is also recommended to repeat karyotype analysis every 10 passages or equivalent population doublings, to prove that hPSCs have diploid karyotype through prolonged culture time [39].

#### 3.2.3. Whole-Genome Analysis

Whole-genome or exome sequencing has been proposed for hPSCs quality control [52,53], but that kind of data is demanding for both data analysis and the interpretation of found variations. One of the main reasons for such testing is the assessment of the risk of tumorigenicity; therefore, focusing on cancer-related genes might be valuable.

#### 3.2.4. Single Nucleotide Polymorphism

Single nucleotide polymorphism (SNP) arrays are based on probe binding and can detect changes in a genome with higher resolution (kb) than G-banding (3–20 Mb). This method enables us to study copy number variations (CNVs), >20% mosaicism and loss of heterozygosity (LOH) but on the other hand, it is unable to detect <20% mosaicism, balanced chromosomal translocations, and inversions [30]. Therefore, it is recommended to combine SNP array with karyotype analysis as it can provide information about balanced chromosomal aberrations [54].

#### 3.2.5. Array CGH

Array comparative genomic hybridization (array CGH) compares two genomes while one of them serves as a reference. Array CGH allows detecting unbalanced genomic changes, including CNVs, with kb resolution. However, this method is unable to detect balanced chromosomal aberrations and low-level mosaicism [55]. On one hand, this method provides information about changes in a genome with higher resolution; however, on the other hand, the interpretation of CNVs is a big challenge, as many clinical consequences of CNVs are not known yet [56]. As array CGH cannot detect balanced chromosomal aberrations it is recommended to combine this assay with karyotype analysis [54].

Ye et al. used CytoSure™ ISCAv2 8×60K Comparative Genomic Hybridization (CGH) microarrays (Oxford Gene Technology) to perform array CGH for the genetic screening of clinical-grade hESC lines and identified approximately 1 Mb gain of chromosome 20q11.21 in two hESC lines. No plausibly pathogenic genetic variations were identified in the other 5 tested hESC lines [43].

#### 3.2.6. Cancer Predisposition Testing

There is a serious risk that potentially harmful mutations in cancer-associated genes, for example in *TP53*, can be transferred to a patient during cell therapy [29]. These mutations could either be present in source cells or acquired during a culture. Sequencing panels of known tumor suppressor genes and proto-oncogenes are available [57,58], and some are already used in a clinical setting. Originally meant for the evaluation of cancer patients, these panels could be repurposed for the quality control of hPSCs, offering high sensitivity in feasible processing. It is highly recommended to test hPSCs for the presence of these mutations, thus increasing the safety of cell therapy.

#### 3.2.7. Residual Vector

Some reprogramming methods use foreign DNA during derivation of hiPSCs. In these cases, vector clearance should be tested. Some of the reprogramming factors have tumorigenic potential, therefore it is desirable from a safety perspective to ensure that the hiPSCs are free of introduced sequences. A quantitative PCR assay with primers detecting vector sequences is a convenient method for vector clearance testing [34].

#### 3.2.8. HLA Typing

Human Leukocyte Antigen (HLA) typing provides useful information about polymorphisms in human genome associated with immune response during transplantations. The results of this assay can connect specific donors with compatible recipients in a way which minimizes the risk of transplant rejection. The histocompatibility of hPSC lines can be compared with a public HLA allele frequency database [59] to find the percentage coverage of the target population.

There is an effort to create HLA-haplotype banks for hiPSCs and hESCs, as they would provide HLA-compatible cells for clinical usage. It was estimated that a bank with 50 HLA homozygous lines would cover 90.7% of the Japanese population for a three-locus match [60]. Similarly, 150 consecutive donors would provide a full match for a minority of recipients (<20%) for the UK population [15]. In comparison, only 10 HLA homozygous donors would provide a complete match for 37.7% of recipients in the UK population.

Services of external laboratory [42] or in-house assays [33,43] were used for the HLA typing when clinical-grade hESC lines were characterized.

### 3.3. Differentiation Assays of hPSCs

To benefit from hPSCs, it is crucial to ensure that the cells are able to leave the stem cell state and form differentiated cell types. There are different approaches to assess the differentiation capacity of a hPSC line.

#### 3.3.1. Embryoid Bodies Formation

The most straightforward method is spontaneous differentiation. When cultured as suspension in absence of FGF, hPSCs tend to form dense structures composed of multiple cell types called embryoid bodies (EBs) [61]. After given time in culture, EBs are harvested and analyzed for the presence of three germ layers. Typically, hPSCs are cultured in low adherence conditions, either U-shaped 96-well plates or hanging drops, in hESC media without FGF. After 1 week, they can be transferred on coated coverslips where they attach, and after another week they can be fixed and stained for various differentiation markers and analyzed by immunocytochemistry. The attachment step can be omitted if EBs are cut and cross-sections are analyzed. The most commonly used markers are beta-III tubulin for ectoderm, alpha-fetoprotein or SOX17 for endoderm and smooth muscle actin for mesoderm [33,34,37]. EBs formation is time and cost-efficient and mirrors the tendencies of a hPSC line to differentiate into one germ layer more than the others. However, the assay is hard to analyze, since an EB is a 3D mixture of many cell types and quantification in immunocytochemistry is hard to design, resulting in vague specification [33,34,62].

#### 3.3.2. Directed Differentiation

Another option to assess the ability to form all three germ layers is the directed differentiation of hPSCs [63]. In a research setting, kits are available for a rapid and straightforward workflow. hPSCs are exposed to differentiation media specific to each germ layer and then analyzed by immunocytochemistry. These methods are very time effective, as the goal is not a specific fully mature cell type, but a mix of precursors from given lineage. Such differentiation only takes a few days. The samples after differentiation are more homogenous and usually in a monolayer, which makes the assessment easier, but even those kits do not expect a pure population of one cell type. Instead of the available kits, in-house method of differentiation can be used, possibly yielding specific cell types, but the time and the price of the process can go up [64]. To our knowledge, there is no consensus on a set of protocols for such differentiation assessment. While truly robust differentiation protocols are scarce, if the facility producing hPSCs already have protocols for directed differentiation at their disposal (for example, if they want to take another step and also produce hPSCs-derived differentiated products) these can also be beneficial for the quality control of a hPSC line.

Both EBs formation and directed differentiation can be further evaluated through expression analysis, which provides more specific data than immunocytochemistry [65]. This assay has been commercialized by Thermo Fisher Scientific as the TaqMan hPSC Scorecard Assay, and offers not only a prepared set of assays, but also access to analysis software for easy interpretation.

#### 3.3.3. Teratoma Formation

Traditionally, in the hPSCs field, the method considered most stringent was teratoma formation [66]. The test lies in injecting undifferentiated hPSCs into an immunocompromised mouse. After several weeks, hPSCs form tumors which are histologically analyzed for tissues of all three germ layers. The test has several drawbacks. From an ethical aspect, the use of an experimental animal should always be thoroughly considered and only accepted when there are no substitutions. Furthermore, the test is very time-consuming as the tumor needs the time to grow, and an animal facility is needed. While teratoma formation is by many considered the gold standard for assessing pluripotency, it has been shown that the methodology is usually poorly specified which makes a comparison of the method results between laboratories virtually impossible [67]. Furthermore, the reproducibility of a standard teratoma assay is quite low, although it can be enhanced using matrices and high levels of injected stem cells [68,69].

### 3.4. Sterility Testing—Bacteria, Endotoxins, Mycoplasma, Viruses

Any product intended for clinical use must undergo sterility testing. As the process of establishing hPSCs line includes cell manipulations and prolonged culture in vitro and there is no method of sterilization without destroying the product, testing of the product is crucial and pre-testing of the starting biological material can prevent wasting resources. Pre-testing possibilities differ in hESCs and hiPSCs. When deriving hESCs, the amount of material is limited and so is the possibility to pre-test. Parents pre-testing is routinely performed by IVF clinics, and sterility and mycoplasma testing can be done from media in the early stages of the process. Establishing hiPSCs can be advantageous, as in addition to pre-screening the donor, there is an option to take a sample from the obtained material and freeze the rest until the test results are ready.

While there can be differences in local pharmacopoeias, there is a set of tests that needs to be performed on the product to ensure its safety in relation to contamination by bacteria, fungi or viruses. Bacterial contamination should be tested by culture-based or broth-based methods for both aerobic and anaerobic bacteria. Testing for mycoplasma can be performed using culture-based methods, but a PCR-based method is also accepted by some authorities [39]. Endotoxin levels are tested by LAL (limulous amoebocyte lysate) assay. Acceptable levels are given by local pharmacopeia, but limits of 5 EU/mL [33,42] or 0.5 EU/mL [34] have been used in hPSCs. A set of tests for human adventitious agents depending on local regulations is required and, where any animal-origin material was used during production, must be accompanied by appropriate non-human adventitious agents testing.

### 3.5. Morphology

Culture conditions may cause differences in cell and colony morphology. There are various surfaces and feeder cells that have an impact on the shape and size of colonies, therefore it is appropriate to have images from every important step of hPSC line establishment and further manipulation.

Undifferentiated colonies of hPSCs have clear borders with well-defined edges. Colonies are typically flat, contain small round cells with large nuclei with prominent nucleoli [70,71]. This morphology is typical for feeder dependent culture and may differ on various surfaces [72,73].

### 3.6. Viability

Viability testing provides useful information about the current state of the culture, but it should not be taken as a quality indicator of hPSCs. It is necessary to validate the chosen method and to measure viability in determined timepoints. It is recommended to not measure viability immediately after thawing as it can provide misleading information.

Flow cytometry was used for measurement of viability in the establishment of clinical-grade hESC lines [42] and for the establishment of hiPSCs [74]. Release criterium for viability was above 50% viable cells per vial in the case of hiPSCs characterization under cGMP conditions in an article published by Baghbaderani [34]. More strict release criteria (>60% viable cells) were used for the establishment of a clinical-grade line of hESCs [42].

It is also recommended to record the number of passages, although the population doubling level can provide more accurate information about cell line age.

## 4. Overview of Characterization Methods

The characterization of hPSCs is a complex process with many phases. After the early phase (EP) of the establishment of the hPSC line it is necessary to create master cell bank (MCB), which can then be used to make a working cell bank (WCB). During the manufacture, in-process testing is recommended in order to prevent wasting resources. Final product (WCB) is then tested for fulfilment of release criteria. We divided the test into two sections, mandatory tests with release criteria (Table 4) and tests “for information only” (Table 5) that are not strictly required but are highly recommended as they offer additional information on the functionality and safety of a hPSC line. Furthermore, we suggest test intervals for each method. Test intervals need to be revised and adjusted in accordance with the risk assessment of the manufacturer.

## 5. Conclusions

hPSCs are a powerful technology with tremendous potential in the clinic. Multiple clinical-grade hPSC lines have been established and clinical trials are ongoing. Nevertheless, there are still obstacles in the translation of hPSC technology to the clinic. One of them is that consensus about the standards for quality control of clinical-grade hPSCs is still not completely reached, which limits not only the comparability of the cell lines but also the credibility of data obtained by preclinical and clinical studies. In this review, we summarize specifications that enable the easier transfer of hPSCs to clinical practice; therefore, we directly help cGMP facilities fulfill the minimal criteria for the establishment of clinical-grade hPSC.

## Figures and Tables

**Table 1:** Summary of main EU directives and guidelines for clinical-grade hPSC line establishment.

Directive/Regulation	Title
2004/23/EC	Standards of quality and safety for the donation, procurement, testing, processing, preservation, storage and distribution of human tissues and cells
2003/94/EC	The principles and guidelines of good manufacturing practice in respect of medicinal products for human use and investigational medicinal products for human use
EudraLex—Volume 4	“The rules governing medicinal products in the European Union” contains guidance for the interpretation of the principles and guidelines of good manufacturing practices for medicinal products for human and veterinary use

hPSC – human pluripotent stem cell.

**Table 2:** Summary of release criteria for pluripotency markers detected by flow cytometry used for the establishment of hPSC line according to cGMP.

hPSCs	Oct3/4	SSEA3	SSEA4	TRA-1-60	TRA-1-81	Sox2	Nanog	AP (TRA-2-54)	SSEA1	CD34	Source
hESCs	-	≥50%	-	≥50%	≥50%	-	-	≥50%	≤50%	-	Tannenbaum [33]
hESCs	>65%	-	>65%	>50%		-	-	-	<15%	-	De Sousa [42]
hiPSCs	>70%	-	>70%	>70%	>70%	-	-	-	-	<5%	Baghbaderani [34]

AP—alkaline phosphatase; - not tested.

**Table 3:** Summary of release criteria for pluripotency markers detected by immunocytochemistry used for the establishment of hPSC lines according to cGMP.

hPSCs	Oct3/4	SSEA3	SSEA4	TRA-1-60	TRA-1-81	Sox2	Nanog	AP (TRA-2-54)	SSEA1	Source
hESCs	≥60%*	≥60%*	≥60%*	≥60%*	≥60%*	-	≥60%*	-	-	Tannenbaum [33]
hESCs	≥70%	-	≥70%	≥70%	≥70%	≥70%	≥70%	-	NA	Ye [43]

NA—no release criterium available, AP—alkaline phosphatase; *colonies; - not tested.

**Table 4:** Summary of mandatory methods used for the characterization of hPSCs for clinical use.

Characteristics	Method	Release Criteria	Test Interval
Differentiation	Embryoid bodies formation/directed differentiation	detection of endoderm, mesoderm and ectoderm	MCB, WCP
Genetic stability	Karyotype analysis	normal diploid (>20 metaphases)	EP, WCB, every 10th passage
Identity	STR analysis	hiPSCs: identical with donor and over time; hESCs: identical over time	hiPSCs: donor cells, WCB
			hESCs: EP, WCB
Vector clearance*	PCR	negative	MCB, WCB
Morphology	Photography	normal morphology**	Continuously
Pluripotency	Flow cytometry	>70% for at least 2 surface and 2 intracellular markers; <10% for SSEA1	EP, MCB, WCB, every 10th passage
Sterility	Endotoxin testing	negative	WCB
	Mycoplasma testing	negative	hiPSCs: donor cells, WCB
			hESCs: EP, WCB
	Adventitious agents	negative	hiPSCs: donor cells, WCB
			hESCs: donors, WCB
	Bacterial contamination	negative	hiPSCs: donor cells, WCB
			hESCs: EP, WCB
Viability	Viability	>60%	EP, MCB, WCP, every 10th passage

EP – early phase, MCB – master cell bank, WCB – working cell bank; *only hiPSCs when applicable ** Colony: flat, clear borders with well-defined edges; cells: small, round with large nuclei with prominent nucleoli.

**Table 5:** Summary of methods for information only used for the characterization of hPSCs for clinical usage.

Characteristics	Method	Test interval
Differentiation	Teratoma formation	WCB
Genetic stability	SNP analysis	WCB
	Whole-genome sequencing	WCB
	Cancer predisposition testing	WCB
Histocompatibility	HLA typing	Dependent on application*
Pluripotency	Immunocytochemistry	EP, MCB, WCB
	Alkaline phosphatase	EP, MCB, WCB
Pluripotency/Differentiation	Transcriptome analysis	WCB

* When establishing hiPSC and hESC banks for HLA-matched tissue, transplantation donor testing or EP is advantageous.

## Abbreviations

cGMP	current good manufacturing practices
CNVs	copy number variations
EBs	embryoid bodies
EP	early phase
hESC	human embryonic stem cell
hiPSC	human induced pluripotent stem cell
hPSC	human pluripotent stem cell
ISCF	International Stem Cell Forum
ISSCR	International Society for Stem Cell Research
IVF	in vitro fertilization
MCB	master cell bank
SOP	standard operating procedures
STR	short tandem repeat
WCB	working cell bank
